# Influence of Hafnium Addition on the Microstructure, Microhardness and Corrosion Resistance of Ti_20_Ta_20_Nb_20_(ZrMo)_20−x_Hf_x_ (where x = 0, 5, 10, 15 and 20 at.%) High Entropy Alloys

**DOI:** 10.3390/ma16041456

**Published:** 2023-02-09

**Authors:** Karsten Glowka, Maciej Zubko, Sandra Gębura, Paweł Świec, Krystian Prusik, Magdalena Szklarska, Danuta Stróż

**Affiliations:** 1 Institute of Materials Engineering, University of Silesia in Katowice, 75 Pułku Piechoty 1A St., 41-500 Chorzów, Poland; 2 Department of Physics, Faculty of Science, University of Hradec Králové, Rokitanského 62, 500-03 Hradec Králové, Czech Republic

**Keywords:** high entropy alloys, multi-component alloys, microstructure, mechanical properties, corrosion resistance

## Abstract

The presented work aimed to investigate the influence of the hafnium/(zirconium and molybdenum) ratio on the microstructure, microhardness and corrosion resistance of Ti_20_Ta_20_Nb_20_(ZrMo)_20−x_Hf_x_ (where x = 0, 5, 10, 15 and 20 at.%) high entropy alloys in an as-cast state produced from elemental powder and obtained via the vacuum arc melting technique. All studied alloys contained only biocompatible elements and were chosen based on the thermodynamical calculations of phase formation predictions after solidification. Thermodynamical calculations predicted the presence of multi-phase, body-centered cubic phases, which were confirmed using X-ray diffraction and scanning electron microscopy. Segregation of alloying elements was recorded using elemental distribution maps. A decrease in microhardness with an increase in hafnium content in the studied alloys was revealed (512–482 HV1). The electrochemical measurements showed that the studied alloys exhibited a high corrosion resistance in a simulated body fluid environment (breakdown potential 4.60–5.50 V vs. SCE).

## 1. Introduction

Up to now, the significant development of engineering materials has contributed to a considerable increase in the number of manufactured and studied materials applied in various industries as well as in everyday life. Additionally, increasing knowledge about the correlation between the physical and chemical properties of elements in the periodic table can further the manufacturing methods, contributing to the production new materials or modification of conventional alloys. The manufacturing method directly determines the structure and properties of the produced materials. Methods such as powder metallurgy or mechanical alloying could contribute to obtaining materials, for example, with controlled grains sizes (TiTaZr gradient porosity materials) or refined grain structures to improve mechanical properties of alloys (NiCoMnIn magnetic shape memory alloys) [1,2,3]. It has been revealed that novel materials containing more than two alloying elements, such as ternary, quaternary, etc., alloys, also exhibit single-phase structures and better properties, i.e., mechanical properties, compared to conventional binary materials [4]. The investigation of these engineering materials led to the discovery of multi-principal element (MPEAs) alloys, containing at least five alloying elements in their structures [5]. High entropy alloys (HEAs), as a novel group of multi-elemental engineering materials, could be excellent candidates for MPEAs. High entropy materials, as a new concept of engineering materials, were first published in the literature in 2004 by Cantor et al. [6].

In the same year, two definitions of high entropy alloys were provided for the first time [7]. HEAs can be defined based on their chemical composition and configurational entropy (ΔS_conf_). High entropy alloys can be defined as materials containing at least five chemical elements in equimolar or near equimolar ratios. Configurational entropy criteria define HEAs as materials that exhibit configurational entropy ΔS_conf_ > 1.61·R (where R is the gas constant) whether they are single-phase or multi-phase at room temperature. It is worth emphasizing that both definitions are in agreement, because a ΔS_conf_ of 1.61·R corresponds to a five element, equiatomic, high entropy material. HEAs also exhibit four characteristic core effects: the high entropy effect, severe lattice distortion effect, sluggish diffusion effect and cocktail effect [8].

Engineering materials for medical applications must achieve strict requirements closely correlated with contact with human tissues and body fluids. The chemical composition of biomedical alloys must contain only a particular group of chemical elements, better known as biocompatible. Furthermore, biomaterials must exhibit a high level of corrosion resistance, preventing ions from being released into the human body [9]. A chemical composition containing only biocompatible elements is one of the strict requirements of biomedical alloys. It was reported that for pure Hf, the biomedical ability of this element was underlined as unknown. However, with increasing knowledge about the biocompatible properties of pure hafnium, it was confirmed that Hf exhibits a high level of corrosion resistance and good tribocorrosion and biocompatibility [10,11,12,13]. Moreover, to our knowledge, Hf-containing titanium-based alloys are also widely used as medical implants [14,15,16,17].

The literature data revealed that the most widely described and studied group of bio-HEAs includes five elemental TiTaNbZrMo alloys [18,19,20,21].

However, Hf-containing, high entropy bioalloys have also been reported in the literature. Long-term corrosion resistance studies described by Gurel et al. for TiTaHfNb, TiTaHfNbZr and TiTaHfMoZr bio-HEAs revealed the high corrosion resistance of the studied alloys due to the presence of Zr and Nb in the microstructure [22]. Additionally, a long-term application could be ensured by passive layers present on the surfaces of studied alloys [22]. An independent study of two biomedical high entropy alloys, TiZrTaHfNb and Ti_1.5_ZrTa_0.5_Hf_0.5_Nb_0.5_, revealed the presence of single-phase BCC structures with different lattice parameters [23]. A high yield strength, differences in hardness and a reduced Young’s modulus were observed for Ti-enriched compositions. Both studied high entropy alloys also exhibited good corrosion resistance but smaller wettability than the Ti6Al4V alloy [23]. Yuan et al. proposed equimolar TiZrHfNbTa for biomedical applications due to its good corrosion resistance measured in Hanks’ solution, which is correlated with the presence of stable oxide layers in comparison to the Ti6Al4V alloy [24]. Yuan et al. reported that TiZrHfNbTa alloys exhibit a unique combination of a low modulus, a good mechanical biocompatibility and a low magnetic susceptibility (similar to pure Zr) [25]. The literature data also reported five elemental Hf-containing TiZrHfNbTa bio-HEAs as promising candidates for biomedical applications due to the presence of the single-phase BCC structure, a high compressive yield strength and plasticity, bio-corrosion resistance, initial cell adhesion and viability in comparison to conventional biomedical alloys [26].

Based on the above-described literature data of biomedical high entropy alloys and biocompatibility of pure Hf, the presented study aimed to design novel Ti_20_Ta_20_Nb_20_(ZrMo)_20−x_Hf_x_ (where x = 0, 5, 10, 15 and 20 at.%) high entropy alloys for possible biomedical applications. The chemical composition of all studied HEAs contains biocompatible chemical elements (Ti, Nb, Ta, Zr, Mo and Hf). The presented work aimed to determine the influence of the Hf/(ZrMo) ratio on the phase formation, microstructure, mechanical properties and corrosion resistance in a simulated body fluid environment. In the chemical composition of all studied alloys, TiTaNb atomic ratios were unchanged due to the high biomedical ability reported in the literature for ternary TiTaNb alloys. The investigation of the influence of Hf addition could significantly develop the current state of knowledge and fill the knowledge gap about six-elemental TiTaNbZrMoHf high entropy alloys. In the presented work, all studied high entropy alloys were fabricated from elemental powders and compared to HEAs reported in the literature with similar chemical compositions, mainly produced from bulk chemical elements, which also underlines the novelty of the presented investigation.

## 2. Materials and Methods

The chemical compositions of all studied Hf-containing high entropy alloys were designed based on the literature reported thermodynamical calculations to predict phase formation after the solidification process. Thermodynamical calculations were carried out to verify whether the studied HEAs met the high entropy material criteria. In the calculation process, the atomic size mismatch differences (differences in atomic radii) of all alloying elements (denoted as δ), the mixing enthalpy (ΔH_mix_), Pauling’s electronegativity (Δχ) and the valence electron concentration (VEC) of all chemical elements were considered. However, long years of studies have revealed that melting temperatures of the alloying elements ((Tm)_i_) should also be considered in the solid solution process. In the literature, the thermodynamical parameter, Ω, described by Zhang et al. could be used [27,28].

Our previous work presents the description of the aforementioned thermodynamical parameters calculations. The physical and chemical properties of all alloying elements in the studied materials have also been described [28,29]. The calculated thermodynamical parameters for all studied Hf-containing HEAs are collected in Table 1.

Concluding from the presented results, all studied HEAs meet the criteria for high entropy materials. Moreover, thermodynamical calculations predicted the formation of multi-phase structures after the solidification process. It was also revealed that with increasing Hf addition, the atomic size mismatch (δ), Pauling’s electronegativity (Δχ) and the valence electron concentration (VEC) decreased. On the other hand, the values of mixing enthalpy (ΔH_mix_) and mixing entropy (ΔS_mix_) increased with the increase in Hf content in all studied bio-HEAs. It was also noted that calculations of the VEC parameter predicted the formation of BCC solid solutions for all studied high entropy materials. Moreover, the Zhang parameter, Ω, for the studied Hf-containing alloys indicated the formation of a multi-phase structure after solidification. It should also be emphasized that the calculation of thermodynamical parameters was in good agreement with X-ray diffraction (XRD) phase analysis and SEM microstructure analysis presented in the current work (later in the text). Due to that, thermodynamical calculations could be crucial in designing the chemical composition process of high entropy materials.

For fabrication of the investigated HEAs, Nb, Ta, Ti (99.9% purity), and Mo (99.5% purity) (Kamb Import-Export) (Warsaw, Poland) and Zr (purity 99.5 %) (Atlantic Equipment Engineers) (Upper Saddle River, NJ, USA) were used for the air plasma spray (APS) technique. The particle size for the elemental powders stated by the manufacturers was as follows: Nb (particle size in the range 70–180 μm), Ta (particle size < 100 μm), Ti (particle size < 90 μm), Mo (particle size < 90 μm) and Zr (particle size < 250 μm). Hf powder (average particle size 98 (56) μm) was produced by mechanical grinding of a bulk rod (diameter d = 15 mm and purity > 99.9%). Elemental powders were precisely weighed using a Radwag AS 60/220/C/2 laboratory balance (Radom, Poland). The homogeneity of elemental particles was achieved by blending for 72 h by our self-designed blending machine. The as-blended powders were compressed under the pressure of 8 tons to form 10 mm green compacts. The as-pressed green compacts of the studied high entropy materials were melted in an Ar protective atmosphere (chamber pressure of 1.2 bar) using the arc melting (AM) technique. A Ti getter of high purity was used to capture the residual gases presented in the arc-melting chamber. The homogeneity of the chemical compositions was ensured by preliminarily melting and mixing for 120 s in a liquified state. Moreover, the received AM samples were overturned, re-melted four times and homogenized for 60 s in a liquified state. It must be underlined that all manufactured HEAs were investigated in an as-cast state. Melted AM buttons were embedded into conductive resin and ground utilizing grinding papers (SiC, grit 320 to 2400) to form metallographic specimen samples (Metkon Forcimat 1 V grinding-polishing machine, equipped with an automatic header Metkon Forcipol (Metkon, Bursa, Turkey)). Diamond suspensions with 6 μm to 1 μm particle sizes were used for further polishing. Finishing was performed using a colloidal silica oxide (SiO_2_) suspension with a particle size of 0.04 μm.

A Panalytical Empyrean diffractometer (Malvern Instruments, Malvern, UK) was used to record X-ray powder diffraction (XRD) patterns. The diffractometer was equipped with a Cu anode (1.54056 Å wavelength) operating at 30 mA electric current and 40 kV voltage. A solid-state, hybrid, ultra-fast, PIXcell^3D^ X-ray detector (Malvern Instruments, Malvern, UK) was used. X-ray diffraction patterns were collected in an angular range of 2θ = 20–140° with 0.026° steps using the θ–θ scan technique (Bragg–Brentano geometry). All measurements were performed at T ≈ 300 K (room temperature). Powley refinement was performed using the FullProf program suite [30].

A JEOL JSM-6480 (JEOL Ltd., Tokyo, Japan) scanning electron microscope (SEM) was employed for microstructure analyses. SEM microphotographs were collected under a 20 kV accelerating voltage. The microscope was additionally fitted with an X-ray spectroscopy energy-dispersive (SEM-EDS) IXRF detector (IXRF, Austin, TX, USA).

Before the electrochemical measurements, the high entropy disc-shaped samples were ground with grinding paper with a gradation of 800–2500 and polished with a colloidal silica oxide (SiO_2_) suspension. The research apparatus was a Metrohm/Eco Chemie Autloab PGSTAT30 Potentiostat/Galvanostat Electrochemical System (Metrohm, Herisau, Switzerland) equipped with a three-electrode electrochemical cell. One of the electrodes, the working electrode (WE), was the investigated material. The counter electrode (CE) was made of platinum. The reference electrode (RE) was a saturated calomel electrode (SCE) placed in a Luggin capillary. The tests were carried out in Ringer’s solution (8.6 g/L NaCl, 0.3 g/L KCl, 0.48 g/L CaCl × 6H_2_O) de-aerated with argon (Ar, 99.999%) at 37(1) °C. The samples were depassived at −1.2 V vs. SCE for 10 min and then tested using the following methods: open circuit potential (E_OC_), registered for 2 h; potentiodynamic polarization (sweep rate of v = 2 mV s^−1^); and electrochemical impedance spectroscopy (EIS). The EIS measurements were carried out at E_OC_ with 10 frequencies per decade scanned using a sine-wave amplitude of 10 mV and a frequency range of f = 50 kHz^−1^ mHz.

A MicroVickers tester 401MVD (Wilson Instruments, Massachusetts, MA, USA) was used in order to perform microhardness measurements. The equipment was fitted with a Vickers tip in the shape of a ~136° pyramid. Measurements were carried out under the load of 1 kN (HV 1) and a dwell time of 10 s.

## 3. Results and Discussion

### 3.1. XRD Phase Analysis of Studied High Entropy Alloys

**Figure 1:**
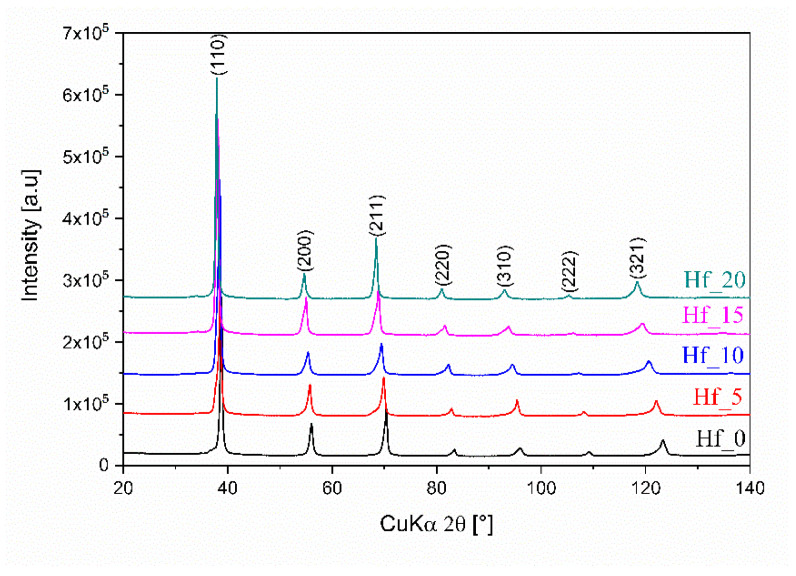
Collected X-ray diffraction patterns for all studied Hf-containing high entropy alloys.

Dual BCC-BCC phases were revealed for all studied Hf-containing high entropy alloys based on performed X-ray diffraction measurements. Moreover, as mentioned above, the presence of two phases corresponds to the dendritic and interdendritic microstructures recorded on SEM images (further in the text). XRD phase analysis also revealed small differences in unit cell parameters of BCC1 and BCC2 phases for the studied high entropy materials. Additionally, during phase analysis, no additional diffraction peaks were observed. Collected XRD patterns for all investigated HEAs are presented in Figure 1.

**Figure 2:**
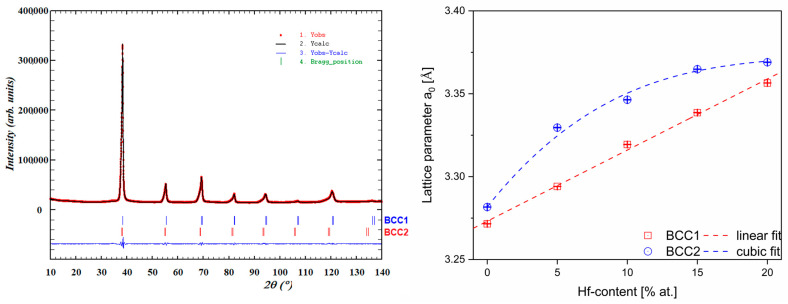
Results of performed Powley refinement for the Hf_10 sample (left) and the variation in the unit cell parameters (BCC1 and BCC2 phases) with changing Hf content for all investigated HEAs (right). Dashed lines indicate linear and cubic fits to the BCC1 and BCC2 phases, respectively.

Additionally, the Powley refinement was measured to determine the lattice parameters of the BCC1 and BCC2 phases. For all studied HEAs, the refinement of lattice parameters lead to an increase in unit cell parameters with increasing Hf concentration. The increase in lattice parameters by Hf addition could be correlated with the high atomic radii of pure Hf (r_i_ = 1.578 Å [29]) which contributes to the expansion of the unit cell. Additionally, the changes in lattice parameters with an increase in Hf content are shown in Table 2 and presented graphically in Figure 2.

As it can be seen based on the results of the Powley refinement (Figure 2), the unit cell parameters of both phases strongly depend on the chemical composition changes. The BCC1 phase, attributed to the higher diffraction peaks (major phase), shows a linear dependence on the Hf content, whereas the BCC2 phase (minor phase) seems to follow cubic dependence. Solid solution crystal lattice parameters depend mainly on the atomic radii due to the fact that atom substitution does not change the coordination or the relative atom’s position. Therefore, the addition of Hf, which possesses the largest atomic radii from the alloying elements present in the studied alloys, enlarges the unit cell.

### 3.2. SEM Microstructure and EDS Chemical Composition Analysis of Studied High Entropy Alloys

Our previous work elsewhere presented the SEM images of the initial powders used to prepare the studied high entropy alloys [29]. As mentioned above, the thermodynamical parameters of the studied bio-HEAs predicted the presence of multi-phase structures, which is in agreement with the results of the XRD phase analysis. An SEM microstructure analysis of the studied Hf-containing high entropy alloys revealed the presence of dendritic and interdendritic regions, which correspond to the dual BCC phases. The presence of dendritic and interdendritic regions is in good agreement with literature-reported data for Mo- and Hf-containing HEAs such as five-elemental TiTaNbZrMo and six-elemental TiTaNbZrHfMo alloys [18,29].

**Figure 3:**
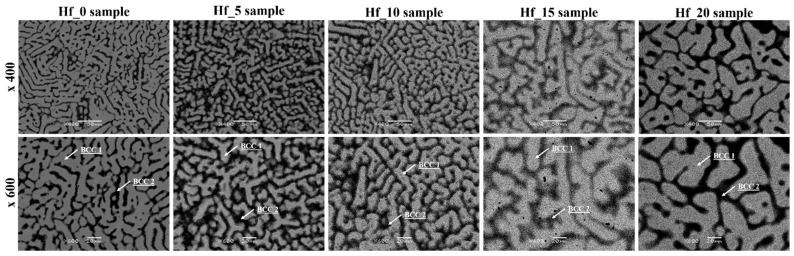
Microstructure images (BSE—backscattered electron contrast) of studied Hf-containing high entropy alloys.

For all studied high entropy alloys, SEM images recorded in backscattered electrons imaging mode (BSE) are presented in Figure 3. The Z-contrast differences between dendritic and interdendritic regions were observed in the recorded microstructure images. As can be seen in the recorded SEM micrograph (see Figure 3) the increase in the Hf alloying element (corresponding to the change in the Hf/MoZr ratio) influences the size of the dendrites. Due to the fact that the dendrites are elongated and interconnected, the average grain size for the studied materials was not determined. Nevertheless, based on a visual consideration, the size of the dendrites increases with the increase in the Hf content. Based on the analysis of the recorded SEM images, the relative amount of phase composition was calculated (see Table 3).

The chemical composition of all studied Hf-containing high entropy alloys was determined using the SEM-EDS technique. For each phase, 40 individual spectra were collected and the average results of the determined chemical composition are presented in Table 3.

The presence of all six alloying elements (with different chemical segregations to the dendritic and interdendritic regions) was revealed by the performed EDS chemical composition analysis. It was shown that for all studied Hf-containing high entropy alloys, the BCC1 phase (corresponding to a dendritic structure) is enriched with Ta, Nb and Mo. However, for the Hf_20 alloy, the BCC1 phase was also Hf-enriched. On the other hand, for the interdendritic structure (BCC2), enrichment in Ti, Nb, Zr and Hf was observed. However, for the Hf_0 sample, the BCC2 phase revealed depletion in Ti. It is worth emphasizing that the chemical composition of the BCC2 phase stays in good agreement with the chemical composition of the BCC2 phase for six-elemental, literature-reported TiTaNbZrHfMo alloys [29]. Literature-reported high entropy materials also revealed an enrichment in Ti, Nb and Zr chemical elements [29]. The segregation of alloying elements in dendritic and interdendritic regions was also observed in literature-described biomedical high entropy alloys, such as five-elemental, Mo-containing TiNbTaZrMo alloys [18,19].

**Figure 4:**
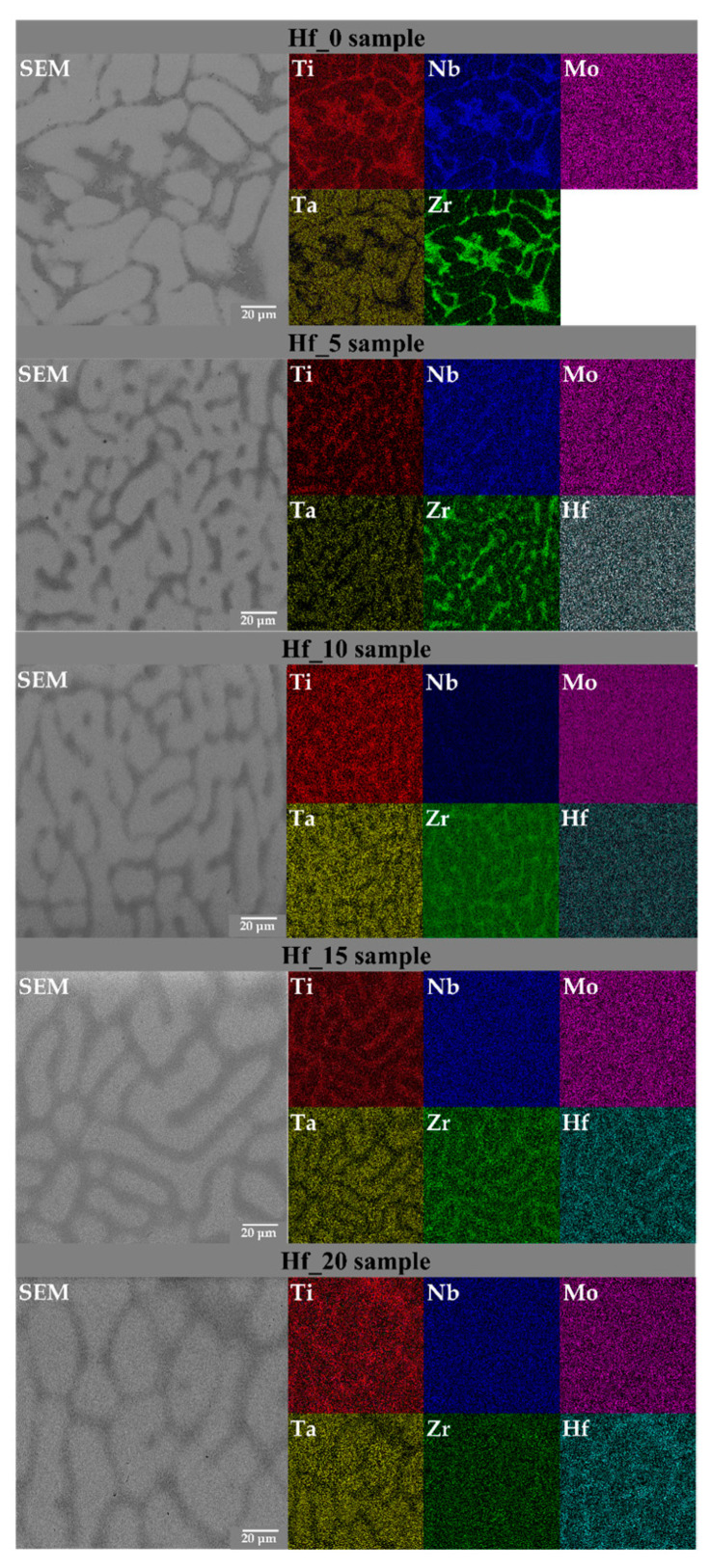
SEM-EDS elemental distribution maps (EDM) images of studied Hf-containing high entropy alloys.

In order to visualize the elemental segregation of alloying elements in the microstructure, SEM elemental distribution maps (SEM-EDM) were collected for all studied samples (Figure 4).

### 3.3. Microhardness of Investigated HEAs

The selected mechanical properties of the investigated Hf-containing high entropy alloys were characterized by means of microhardness measurements. Due to the Vickers tip’s micrometric size, the presented microhardness is an average microhardness for both BCC1 and BCC2 regions. For every sample, 30 indentation measurements were performed, and the obtained result averages are presented in Table 4.

**Figure 5:**
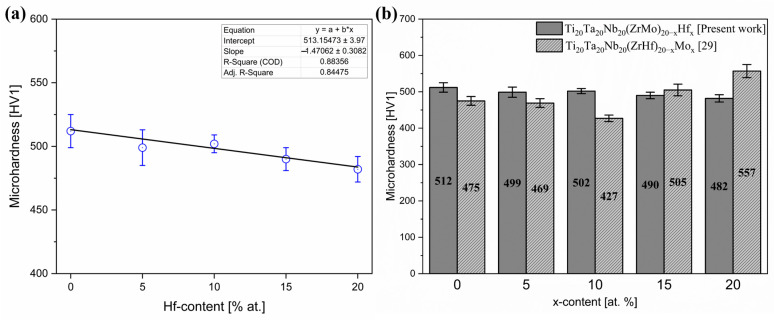
(**a**) Microhardness of studied Hf-containing high entropy alloys with a linear fit, (**b**) comparison of microhardness with literature-reported high entropy alloys.

Moreover, a diagram of the dependence of microhardness on the Hf content in the studied materials is presented in Figure 5a. The obtained microhardness results were compared with our literature-reported Mo-containing high entropy alloys (Figure 5b) [29].

The measured microhardness decreases with the increase in Hf addition. The recorded results indicate linear dependence of alloy microhardness on Hf content. The dependence of hardness on the average grain size is described by the Hall–Petch relation and the grain enlargement provokes a reduction in material hardness. As mentioned earlier, the size of the dendrites seems to increase with the increase in Hf concentration, which corresponds to the microhardness reduction indicated by the performed measurements. The obtained microhardness results were compared with literature-reported biomedical high entropy compositions, conventional biomaterials (Table 5) and previously reported results for Mo-containing HEAs [29]. The results indicate that at up to 10% Hf addition, the microhardness was higher in comparison to Mo-containing HEAs. On the other hand, a further decrease in microhardness was observed for the samples containing up to 20% hafnium.

The microhardness was comparable with the literature-reported six elemental TiTaNbZrHfMo high entropy alloys and conventional 316 L stainless steel after the laser cladding process (467.8 HV1) [29,32]. On the other hand, the microhardness of all studied bio-HEAs was higher than conventional Ti-based alloys, such as Ti6Al4V, titanium grade 4 and commercially pure Ti. Furthermore, the much higher microhardness of Hf-containing materials compared to lamellar bone limits the potential implantation ability of the investigated high entropy alloys.

The differences in microhardness of all studied materials are probably closely correlated to the SEM-EDS chemical compositions of the BCC1 and BCC2 phases. For the Hf_10 sample, the elemental segregations and enrichment in elements exhibiting higher hardness increased the microhardness compared to other studied samples.

### 3.4. Corrosion Resistance Properties of Investigated HEAs

The stabilized values of the open circuit potentials measured for individual samples are summarized in Table 6. The obtained E_OC_ values can be considered as approximate values of the corrosion potential of the studied system. From the obtained results, the influence of the hafnium content on the E_OC_ value is clearly visible. The tendency for E_OC_ values to shift towards positive potentials with increasing Hf content may indicate an improvement in corrosion resistance (Table 6). The self-passive oxide layer and its composition and structure play the main role in corrosion resistance.

**Figure 6:**
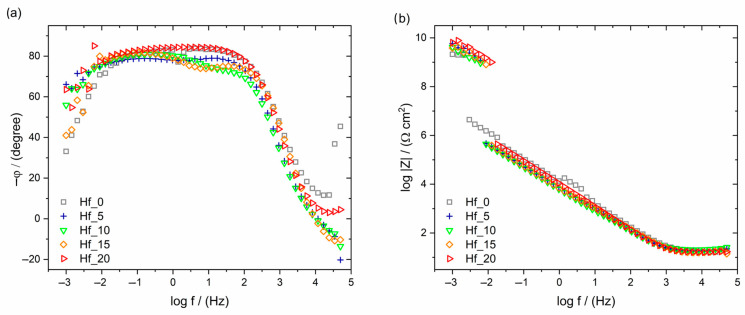
Bode diagram, registered at E_OC_, (**a**) φ = f(log f) and (**b**) log |Z| = f(log f) curves for Ti_20_Ta_20_Nb_20_(ZrMo)_20−x_Hf_x_ electrodes exposed to Ringer’s solution at 37 °C.

EIS results are presented in the form of Bode diagrams in Figure 6. In Figure 6a, one can observe only a one-time constant which indicates the presence of a self-passive oxide layer on the tested HEAs. Additionally, the wide plateau in the frequency range of 0.01–1000 Hz visible in the Bode diagram proves the passive protection of the tested alloys [37].

The values of log |Z|_f→0.01 Hz_ determine the material’s resistance to pitting corrosion. For alloys with hafnium addition, the log |Z|_f→0.01 Hz_ values are similar and a little lower than for alloys without Hf. The results show that the log |Z|_f→0.01 Hz_ values slightly increase with increasing Hf content (Table 6 and Figure 6b). Better resistance to pitting corrosion, which is crucial in biomaterials, is connected with a higher log |Z|_f→0.01_ Hz value. The material’s susceptibility to pitting corrosion could damage the implant surface and weaken its mechanical properties, leading to fracture and the diffusion of corrosion products into the peri-implant tissues, resulting in poisoning and the consequent rejection of the implant [38]. The wider plateau (Figure 6a) and the higher impedance values (Figure 6b) correspond to a more efficient corrosion resistance.

**Table 6:** Open circuit potentials (E_OC_) and the log|Z|_f→0.01Hz_ values registered for studied materials with different Hf contents compared with the literature-reported breakdown potential (E_BD_) for conventional biomaterials.

Sample	E_OC_ vs. SCE (mV)	log|Z|_f→0.01Hz_ (Ω∙cm^2^)	E_BD_ vs. SCE (V)	Reference
Hf_0	−228	6.05	~5.00	Present work
Hf_5	−270	5.54	~4.60	Present work
Hf_10	−199	5.50	~5.50	Present work
Hf_15	−170	5.59	~5.35	Present work
Hf_20	−139	5.64	~5.45	Present work
Ti_20_Ta_20_Nb_20_(ZrHf)_10_Mo_20_	―	―	~6.18	[29]
Ti_20_Ta_20_Nb_20_(ZrHf)_12.5_Mo_15_	―	―	~6.11	
Ti_20_Ta_20_Nb_20_(ZrHf)_15_Mo_10_	―	―	5.57	
Ti15Mo	―	―	5.50	[39]
Ti_20_Ta_20_Nb_20_(ZrHf)_17.5_Mo_5_	―	―	5.15	[29]
TiNbZrTa	―	―	5.00	[40]
Ti15Mo	―	―	4.51	[41]
Ti_20_Ta_20_Nb_20_(ZrHf)_20_	―	―	4.33	[29]
Titanium Grade 7	―	―	2.40	[42]
Ti6Al4V	―	―	1.53	[43]
cp-Ti Grade 2	―	―	1.48	
Ti6Al7Nb	―	―	1.38	
Ti13Nb13Zr	―	―	1.25	
316L stainless steel	―	―	0.96	[44]
Pure Ti	―	―	0.50	[42]
Ti15Nb	―	―	0.45	[45]
NiTi SMA	―	―	0.45	[46]
Ti45Nb	―	―	0.28	[45]

**Figure 7:**
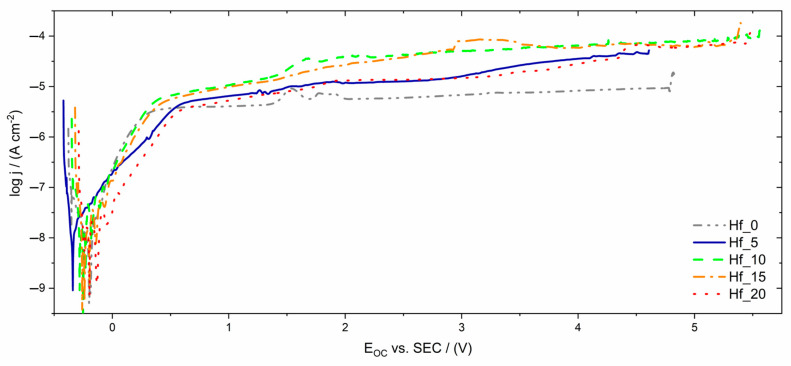
Anodic polarization curves for Ti_20_Ta_20_Nb_20_(ZrMo)_20−x_Hf_x_ electrodes exposed to Ringer’s solution at 37 °C.

The potentiodynamic curves registered for the studied electrodes in Ringer’s solution are characteristic of self-passivating materials (Figure 7). The breakdown potential of the oxide layer on the electrode’s surface varied depending on the Hf content in the studied alloys. The highest breakdown potential, at around 5.5 V vs. SCE, can be observed for samples containing 15–20% hafnium (Figure 7). The lowest value (4.6 V vs. SCE) was registered for sample Hf_5. On the potentiodynamic curves, among the passive range, a slight increase in current density in the potential range 1.5–2 V vs. SCE could be distinguished, which might be connected with oxidation of the nonstoichiometric oxides at increased potential values. It is clearly observed on the potentiodynamic curve registered for Hf_0.

Figure 7 shows the typical potentiodynamic curves of self-passivating materials with a wide passive range. The small increase in current density observed around the potential from 1.5 to 2 V vs. SCE might be caused by oxidation of the nonstoichiometric oxides. The registered breakdown potential of the oxide layer, E_BP_, depends on the Hf content in the studied alloys. The highest breakdown potential value (5.5 V vs. SCE) could be distinguished for samples containing 15–20% hafnium (Figure 7). The E_BD_ value for sample Hf_5 (4.6 V vs. SCE) was the lowest.

The obtained results of corrosion resistance measurements are summarized in Table 6. The determined breakdown potential was also compared with literature-reported high entropy alloys and other conventional biomaterials.

**Figure 8:**
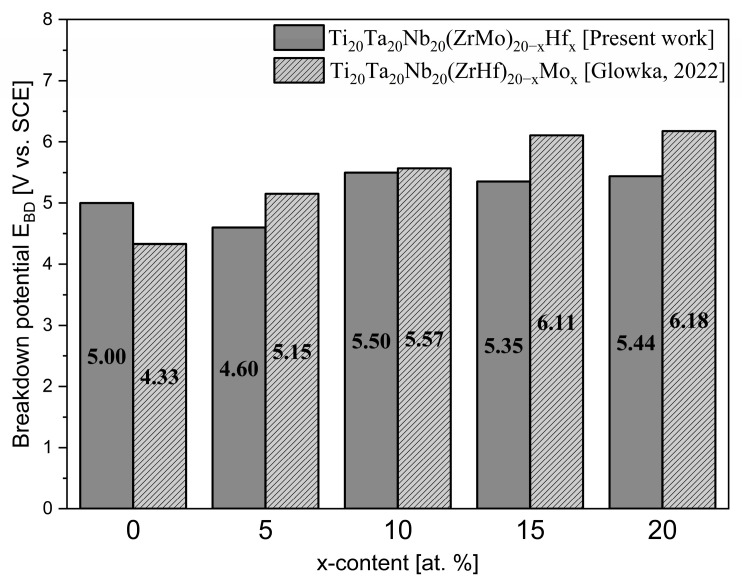
Breakdown potential (E_BD_) of studied Hf-containing alloys in comparison to the literature-reported high entropy alloys [29].

The literature data show that the E_BD_ value oscillates in the range of 0.5–2.4 V for titanium and its alloys, which are commonly used in implantology. The breakdown potentials of the studied alloys were also comparable with literature-reported Ti_20_Ta_20_Nb_20_(ZrHf)_20-x_Mo_x_ (Figure 8) and the Ti15Mo alloy. The E_BD_ values of all studied Hf-containing bio-HEAs were much higher than conventional biomedical materials. When comparing these results, it can be concluded that it is possible to consider the studied alloys as a biomaterial with potential medical applications [42,47].

## 4. Conclusions

Six-elemental HEAs (Ti_20_Ta_20_Nb_20_(ZrMo)_20−x_Hf_x_, where x = 0, 5, 10, 15 and 20 at.%) were fabricated using the arc melting method from powders of chemical elements. The impact of the Hf/(ZrMo) ratio on the microstructure formation, phase composition, corrosion resistance and particular mechanical properties was investigated in the as-cast state. All investigated HEAs were produced from biocompatible elements for further biomedical applications.

For all studied Hf-containing HEAs, the presence of dual BCC phases (dendritic and interdendritic) was observed by XRD and SEM techniques. Additionally, SEM-EDS elemental distribution maps showed the segregations of alloying elements in the microstructure. XRD Powley refinement revealed slight differences in lattice parameters between dendritic and interdendritic phases. An analysis showed that the lattice parameters of the BCC1 phase were linearly dependent on the Hf composition, whereas the BCC2 phase showed cubic dependence. Moreover, the unit cell parameters were in good agreement with the lattice parameters of other HEAs with similar chemical compositions (TiTaNbZrMo or TiTaNbZrHf) already reported in the literature.

Mechanical property measurements confirmed the higher microhardness of all studied Hf-containing high entropy alloys compared to conventional biomaterials such as 316 L stainless steel, Ti6Al4V and cp-Ti alloys. Moreover, a much higher microhardness was measured compared to lamellar bone, which limits the potential application as a bone implant. On the other hand, the microhardness of the investigated materials was similar to the Mo-containing literature-reported Ti_20_Ta_20_Nb_20_(ZrHf)_20−x_Mo_x_ alloys. However, it was shown that Hf addition decreased the microhardness of the studied alloys compared to Mo addition. A further increase in Hf content and a decrease in Mo could probably contribute to the decreased microhardness of the studied alloys.

The electrochemical measurements showed that the studied HEAs were characterized by a high corrosion resistance in a simulated body fluid environment. The breakdown potential oscillated from 4.60 to 5.50 V vs. SCE, comparable with Mo-containing high entropy alloys with a similar chemical composition and binary Ti15Mo widely used in medicine. Furthermore, the studied bio-HEAs exhibited a higher E_BD_ compared to conventional biomaterials, which underlines their high potential for applications in medicine.

## Figures and Tables

**Table 1:** Thermodynamical parameters calculated for the studied Hf-containing HEAs in comparison to literature reported biomedical HEAs with similar chemical composition: δ—atomic size mismatch, ΔH_mix_—mixing enthalpy, ΔS_mix_—mixing entropy, Δχ—electronegativity differences, VEC—valence electron concentration, Ω—Zhang parameter; BCC—body-centered cubic.

Chemical Composition	Abbreviation	δ (%)	ΔH_mix_ (kJ∙mol^−1^)	ΔS_mix_ (J·(mol·K)^−1^)	Δχ (eV)	VEC	Ω
Ti_20_Ta_20_Nb_20_(ZrMo)_20_	Hf_0	5.46	−1.76	13.38	0.282	4.80 (BCC)	19.77 (multi-phase)
Ti_20_Ta_20_Nb_20_(ZrMo)_17.5_Hf_5_	Hf_5	5.42	−1.24	14.35	0.274	4.75 (BCC)	30.21 (multi-phase)
Ti_20_Ta_20_Nb_20_(ZrMo)_15_Hf_10_	Hf_10	5.37	−0.70	14.68	0.263	4.70 (BCC)	54.51 (multi-phase)
Ti_20_Ta_20_Nb_20_(ZrMo)_12.5_Hf_15_	Hf_15	5.29	−0.16	14.72	0.251	4.65 (BCC)	246.85 (multi-phase)
Ti_20_Ta_20_Nb_20_(ZrMo)_10_Hf_20_	Hf_20	5.19	0.40	14.53	0.235	4.60 (BCC)	94.45 (multi-phase)

**Table 2:** Lattice parameters obtained using Powley refinement of the XRD patterns for all studied Hf-containing HEAs.

Sample	Phase	Lattice Parameters a_0_, (Å)
Hf_0	BCC1	3.2716(1)
	BCC2	3.2817(1)
Hf_5	BCC1	3.2941(2)
	BCC2	3.3296(1)
Hf_10	BCC1	3.3194(1)
	BCC2	3.3464(2)
Hf_15	BCC1	3.3386(2)
	BCC2	3.3648(2)
Hf_20	BCC1	3.3565(1)
	BCC2	3.3690(1)

**Table 3:** EDS chemical compositions (at.%) and phase contribution for BCC1 (dendritic) and BCC2 (interdendritic) areas of the investigated HEAs.

Studied Alloy	Elements	Ti	Ta	Nb	Zr	Mo	Hf	Phase Contribution
**Hf_0**	Nominal	20.0	20.0	20.0	20.0	20.0	―	―
	BCC1	12.9(2)	26.2(4)	24.7(4)	8.2(2)	25.5(4)	―	81(8)%
	BCC2	8.4(1)	15.9(4)	20.6(1)	45.6(6)	9.5(3)	―	19(8)%
**Hf_5**	Nominal	20.0	20.0	20.0	17.5	17.5	5.0	―
	BCC1	13.3(2)	23.7(8)	26.9(1)	10.6(6)	21.5(2)	3.9(3)	67(8)%
	BCC2	20.4(1)	7.9(2)	22.2(1)	31.0(3)	10.2(2)	8.4(1)	33(8)%
**Hf_10**	Nominal	20.0	20.0	20.0	15.0	15.0	10.0	―
	BCC1	12.6(1)	28.0(3)	26.0(1)	8.5(2)	18.4(1)	6.5(2)	67(7)%
	BCC2	18.4(1)	11.2(4)	22.0(1)	25.7(3)	10.6(2)	12.2(2)	33(7)%
**Hf_15**	Nominal	20.0	20.0	20.0	12.5	12.5	15.0	―
	BCC1	12.2(1)	26.2(3)	22.5(1)	7.2(1)	15.8(1)	16.0(2)	68(10)%
	BCC2	17.2(1)	10.8(3)	19.1(1)	17.7(2)	10.3(1)	24.9(2)	32(10)%
**Hf_20**	Nominal	20.0	20.0	20.0	10.0	10.0	20.0	―
	BCC1	13.5(1)	28.2(2)	23.1(1)	4.6(1)	13.6(1)	17.0(2)	73(8)%
	BCC2	20.2(1)	12.6(3)	19.0(1)	9.7(1)	9.6(1)	28.9(3)	27(8)%

**Table 4:** Microhardness of Hf-containing studied high entropy alloys.

Sample	Microhardness [HV1]
Hf_0	512 (13)
Hf_5	499 (14)
Hf_10	502 (7)
Hf_15	490 (9)
Hf_20	482 (10)

**Table 5:** Comparison of microhardness of all investigated alloys and literature-reported biomaterials (conventional and HEAs).

Sample	Microhardness [HV1]	Reference
Ti_20_Ta_20_Nb_20_(ZrMo)_20_	512(13)	Present work
Ti_20_Ta_20_Nb_20_(ZrMo)_15_Hf_10_	502(7)	Present work
Ti_20_Ta_20_Nb_20_(ZrMo)_17.5_Hf_5_	499(14)	Present work
Ti_20_Ta_20_Nb_20_(ZrMo)_12.5_Hf_15_	490(9)	Present work
Ti_20_Ta_20_Nb_20_(ZrMo)_10_Hf_20_	482(10)	Present work
Ti_20_Ta_20_Nb_20_ (ZrHf)_10_Mo_20_	557	[29]
Ti_20_Ta_20_Nb_20_ (ZrHf)_12.5_Mo_15_	505	
Ti_20_Ta_20_Nb_20_(ZrHf)_20_	475	
Ti_20_Ta_20_Nb_20_(ZrHf)_17.5_Mo_5_	469	
316L SS (laser cladded)	467.8	[31]
Ti_20_Ta_20_Nb_20_ (ZrHf)_15_Mo_10_	427	[29]
Ti6Al4V (selective laser melted)	390	[32]
Titanium Grade 4 (after SPD process)	330	[33]
Titanium Grade 4 (in an initial state)	170	
cp-Ti (after HPT)	305	[34]
316L SS (additive manufactured)	300	[35]
Lamellar bone	88.8	[36]

## Data Availability

Data sharing not applicable.
